# Mitochondrial genomes of two *Sinochlora* species (Orthoptera): novel genome rearrangements and recognition sequence of replication origin

**DOI:** 10.1186/1471-2164-14-114

**Published:** 2013-02-20

**Authors:** Chunxiang Liu, Jia Chang, Chuan Ma, Ling Li, Shanyi Zhou

**Affiliations:** 1 Key Laboratory of Zoological Systematics and Evolution, Institute of Zoology, Chinese Academy of Sciences, 100101, Beijing, China; 2 College of Life Science, Guangxi Normal University, Guilin, Guangxi, China; 3 Beijing Institutes of Life Science, Chinese Academy of Sciences, Beijing, China

**Keywords:** *Sinochlora longifissa*, *Sinochlora retrolateralis*, Mitochondrial genome, Genome rearrangements, Recognition sequence

## Abstract

**Background:**

Orthoptera, the largest polyneopteran insect order, contains 2 suborders and 235 subfamilies. Orthoptera mitochondrial genomes (mitogenomes) follow the ancestral insect gene order, with the exception of a *trnD*-*trnK* rearrangement in Acridomorphs and rare tRNA inversions. A question still remains regarding whether a long thymine-nucleotide stretch (T-stretch) involved in the recognition of the replication origin exists in the control region (CR) of Orthoptera mitochondrial DNA (mtDNA). Herein, we completed the sequencing of whole mitogenomes of two congeners (*Sinochlora longifissa* and *S. retrolateralis*), which possess overlapping distribution areas. Additionally, we performed comparative mitogenomic analysis to depict evolutionary trends of Orthoptera mitogenomes.

**Results:**

Both *Sinochlora* mitogenomes possess 37 genes and one CR, a common gene orientation, normal structures of transfer RNA and ribosomal RNA genes, rather low A+T bias, and significant C skew in the majority strand (J-strand), resembling all the other sequenced ensiferans. Both mitogenomes are characterized by (1) a large size resulting from multiple copies of an approximately 175 bp GC-rich tandem repeat within CR; (2) a novel gene order (*rrnS*-*trnI*-*trnM*-*nad2*-CR-*trnQ*-*trnW*), compared to the ancestral order (*rrnS*-CR-*trnI*-*trnQ*-*trnM*-*nad2*-*trnW*); and (3) redundant *trnS(UCN)* pseudogenes located between *trnS(UCN)* and *nad1*. Multiple independent duplication events followed by random and/or non-random loss occurred during *Sinochlora* mtDNA evolution. The Orthoptera mtDNA recognition sequence of the replication origin may be one of two kinds: a long T-stretch situated in or adjacent to a possible stem-loop structure or a variant of a long T-stretch located within a potential stem-loop structure.

**Conclusions:**

The unique *Sinochlora* mitogenomes reveal that the mtDNA architecture within Orthoptera is more variable than previously thought, enriching our knowledge on mitogenomic genetic diversities. The novel genome rearrangements shed light on mtDNA evolutionary patterns. The two kinds of recognition sequences of replication origin suggest that the regulatory sequences involved in the replication initiation process of mtDNA have diverged through Orthoptera evolution.

## Background

Insect mitogenomes are generally compact with few intergenic spacers and possess stable gene content and organization. They are usually about 16 kb in size and bear 13 protein-coding genes (PCGs), 2 ribosomal RNA genes (rRNAs), 22 transfer RNA genes (tRNAs), and one control region (CR) that includes replication and transcription origins [1]. However, extensive studies have revealed that gene order rearrangement and size variation that results from the presence of tandem repeats (TRs) and other non-coding regions occur more often than previously expected. Much attention has been paid to studies focusing on unveiling genomic diversities and evolutionary trends. Recently, a mitogenomic investigation of congeneric species has yielded a valuable approach for assessing mtDNA evolutionary trends [2]. Unfortunately, in spite of the large number of insect species, the limited availability of complete mitogenomic sequence data, including those of congeneric species, impedes a thorough understanding of the insect mitogenomes.

Orthoptera, the largest polyneopteran order, contains 235 subfamilies and over 22,500 described species, taxonomically divided into two suborders: Caelifera (locusts and grasshoppers) and Ensifera (katydids and crickets etc.) [3]. Orthoptera mitogenomes generally possess a relatively stable gene content and organization identical to the insect ancestor [1]. Only a *trnD*-*trnK* rearrangement in the lineage Acridomorpha [4,5], inversion of the gene cluster *trnE*-*trnS(AGN)*-*trnN* in *Teleogryllus emma*[6], and occasional inversion of *trnW* in the migratory locust [7] have been discovered. A mitogenomic divergence in the AT-bias between the two suborders has been demonstrated, i.e., the AT-content was generally lower in Ensifera than in Caelifera [4]. Additionally, a possible stem-loop structure has been implicated in mtDNA replication initiation in a few caeliferans [8,9], which contrasts with the recognition of the mtDNA replication origin (O_R_) that involves a long T-stretch in most other insects [8]. However, representative katydid mitogenomes, e.g., *Anabrus simplex* and *Deracantha onos*, revealed the existence of a long T-stretch [10,11]. Furthermore, mitogenomes of 27 Caelifera and 10 Ensifera species available from GenBank demonstrate a distinct taxon sampling imbalance between the two suborders. Thus, additional Ensifera taxon sampling is essential to investigate the mitogenomic genetic diversities and evolutionary trends.

The genus *Sinochlora* Tinkham [12], Chinese bush katydid, belongs to the subfamily Phaneropterinae in the suborder Ensifera. In *Sinochlora*, one species *S. longifissa* is widespread in East Asia including Japan, Korea, and southern China, and two species are widely distributed and indigenous in southern China [13]. Other species are endemic in various large mountains in southern China including low-altitude areas in Tibet [13,14]. Investigation of the mitogenomic evolutionary trends of the genus is helpful to unveil the molecular mechanism of the divergence patterns.

Herein, we chose two representative species, *S. longifissa* distributed in East Asia, and *S. retrolateralis* narrowly endemic in southern China, for mitogenomic investigation. We sequenced the mitogenomes of the two congeners, unveiled novel mitogenomic characteristics, and outlined the possible rearrangement mechanism. Additionally, we also compared the recognition sequences of the O_R_ in known Orthoptera mitogenomes. Overall, we attempted to provide the molecular basis for understanding diversification of the genus *Sinochlora* and depict molecular diversity and evolutionary trends of Orthoptera mitogenomes.

## Results and discussion

### Genome organization

**Figure 1:**
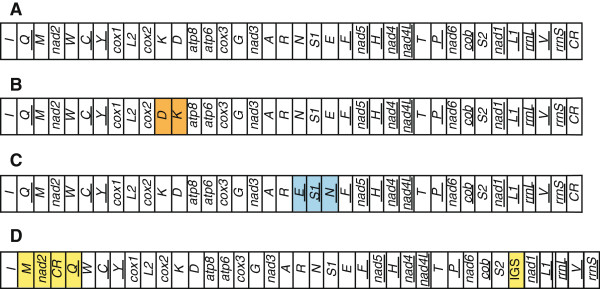
**Mitogenome organization across sequenced Orthoptera.** Genome organization of (**A**) most sequenced ensiferans, few caeliferans, and proposed insect ancestor; (**B**) all sequenced Acridomorphans in Caelifera; (**C**) *Teleogryllus emma*, an ensiferan; (**D**) the two *Sinochlora* species. For the purpose of presentation, the circular mitogenomes are linearized. The translocated regions are highlighted in color. Genes are transcribed from left to right, except those underlined which are transcribed from right to left. Gene lengths are not to scale.

We sequenced the complete mitogenome of *S. longifissa* (18,133 bp) and the nearly complete mitogenome of *S. retrolateralis* (17,209 bp) with a partial CR. The mitogenomes of *S. longifissa* and *S. retrolateralis* have been deposited in the GenBank database under accession numbers of KC467055 and KC467056, respectively. They are currently the largest Orthoptera mitogenomes on GenBank. Their large sizes are due to two large non-coding regions, i.e., the CR and one intergenic spacer (IGS) located between *trnS(UCN)* and *nad1*. Both mitogenomes (Table 1) resemble the available ensiferan mitogenomes [4] and the proposed ancestral insect mitogenome [1] in gene content and orientation, tRNA anticodons, and tRNA/rRNA structures (Figure 1). Additionally, there are several gene overlaps, such as the open reading frames (ORF) of *atp6*-*atp8* and *nad4L*-*nad4*, each of which overlaps by seven nucleotides (Table 1). A few other IGS are also present in the mitogenomes (Table 1).

**Table 1:** **Mitogenome organization of the two*Sinochlora*species**

**Feature**	**Strand**	**Position**		**Initiation codon**		**Stop codon**		**Anticodon**
		***Sl***	***Sr***	***Sl***	***Sr***	***Sl***	***Sr***	
*trnI*	J	1-66(+5)	1-66(+5)					GAT
*trnM*	J	72-138(-3)	72-138(0)					CAT
*nad2*	J	139-1161(0)	139-1161(0)	ATA	ATA	TAA	TAA	
ATR	——	1162-2552(0)	1162-2547(0)					
TRU	——	2553-4281(+33)	2548-3419(+33)*					
*trnQ*	N	4383-4315(+35)	3521-3453(+36)					TTG
*trnW*	J	4419-4484(-1)	3558-3623(-1)					TCA
*trnC*	N	4547-4484(+2)	3687-3623(+3)					GCA
*trnY*	N	4616-4550(+16)	3757-3691(+16)					GTA
*cox1*	J	4633-6148(0)	3774-5289(0)	TCT	TCT	T	T	
*trn L(UUR)*	J	6149-6212(+2)	5290-5353(+2)					TAA
*cox2*	J	6215-6905(0)	5356-6045(+1)	ATG	ATG	T	TAA	
*trnK*	J	6906-6975(-1)	6047-6116(-1)					CTT
*trnD*	J	6975-7042(0)	6116-6183(0)					GTC
*atp8*	J	7043-7207(-7)	6184-6348(-7)	ATC	ATC	TAA	TAA	
*atp6*	J	7201-7875(+2)	6342-7015(0)	ATG	ATG	TAA	TA	
*cox3*	J	7878-8678(0)	7016-7815(0)	ATG	ATG	TAA	TA	
*trnG*	J	8679-8742(0)	7816-7880(0)					TCC
*nad3*	J	8743-9096(+6)	7881-8234(+7)	ATC	ATC	TAA	TAA	
*trnA*	J	9103-9170(-1)	8242-8308(-1)					TGC
*trnR*	J	9170-9232(+4)	8308-8370(+4)					TCG
*trnN*	J	9237-9302(0)	8375-8440(0)					GTT
*trnS (AGN)*	J	9303-9369(0)	8441-8507(0)					GCT
*trnE*	J	9370-9435(-2)	8508-8573(-2)					TTC
*trnF*	N	9496-9434(0)	8634-8572(0)					GAA
*nad5*	N	11213-9497(0)	10351-8635(0)	ATA	ATA	T	T	
*trnH*	N	11278-11214(0)	10416-10352(0)					GTG
*nad4*	N	12614-11279(-7)	11752-10417(-7)	ATG	ATG	T	T	
*nad4L*	N	12907-12608(+1)	12045-11746(+10)	ATA	ATA	TAA	TAA	
*trnT*	J	12909-12972(-1)	12056-12119(-1)					TGT
*trnP*	N	13040-12972(0)	12187-12119(0)					TGG
*nad6*	J	13041-13558(0)	12188-12705(0)	CTG	TTG	TA	TA	
*cob*	J	13559-14693(0)	12706-13840(0)	ATG	ATG	T	T	
*trnS(UCN)*	J	14694-14762(+161)	13841-13908(+94)					TGA
*nad1*	N	15862-14924(+3)	14941-14003(+3)	ATT	ATT	TAG	TAG	
*trnL(CUN)*	N	15928-15866(-1)	15007-14945(0)					TAG
*rrnL*	N	17230-15928(-2)	16307-15008(0)					
*trnV*	N	17300-17229(0)	16378-16308(0)					TAC
*rrnS*	N	18083-17301(+50)	17159-16379(+50)					

*Sl*, *S. longifissa*; *Sr*, *S. retrolateralis*. The number of intergenic nucleotides before the gene starts are shown in parenthesis. The asterisk indicates that the tandem repeat region in *S. retrolateralis* was not completely sequenced.

### Nucleotide composition

**Figure 2:**
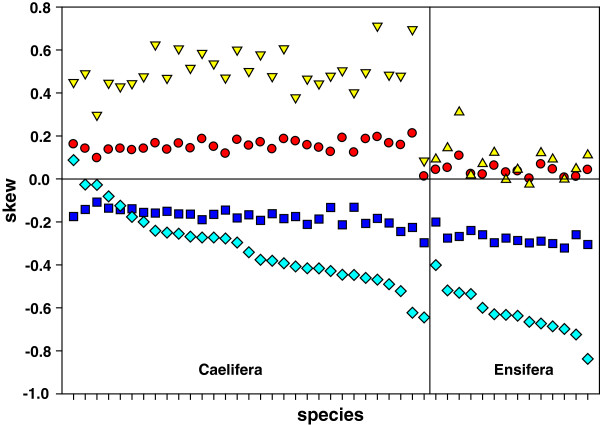
**Comparison of AT and GC skews between Caelifera and Ensifera.** Triangles and diamonds separately represent AT and GC skews at P_4fd_ in protein-coding genes on the J-strand. Circles and squares separately represent AT and GC skews of the whole J-strand.

Like other ensiferans, the two mitogenomes show comparatively low A+T bias (69.05% in *S. longifissa* and 70.08% in *S. retrolateralis*) (Additional file 1), compared with most caeliferans [4]. The AT skew is 0.0017 in *S. longifissa* and 0.0063 in *S. retrolateralis*, while the GC skew is -0.2983 in the former and -0.3218 in the latter, indicating weak A skew and strong C skew in the J-strand. Through comparison of nucleotide skew in all sequenced Orthoptera, the skew divergence between the two suborders was detected. Within the Ensifera, the AT skew values of the whole J-strand range from -0.1 to 0.1 and GC skew values are lower than -0.25, showing the same skew patterns as in *Sinochlora* species (Figure 2). By contrast, within the Caelifera except a tridactylid *Ellipes minuta*[4], absolute values of AT skew and GC skew of the J-strand range from 0.1 to 0.25, indicating pronounced A skew and C skew (Figure 2). The synonymous fourfold degenerate third codon positions (P_4fd_) suffer less selective constraints, and thus could indicate background mutational pressures on nucleotide skew [15]. The skew patterns at the P_4fd_ between the two suborders are consistent with those on the J-strand (Figure 2). It is proposed that nucleotide composition bias and skew are caused by the selection-mutation-drift equilibrium in the molecular evolution [16]. The asystematical directional mutation pressure and corresponding deaminations of cytosine and adenine in the mitogenomes are involved in the mutation processes [17].

### Start and stop codons

All PCGs except *cox1* and *nad6* start with typical ATN codons. A previous study concerning Orthoptera mitogenomes suggested that the *cox1* gene may start with an irregular tetranucleotide codon AUGA [4]. Similar irregular tetranucleotide codons were also proposed as start codons in *Drosophila*[18,19]. However, there is no experimental evidence for the use of a 4-bp start codon in any creature. Recent research on characteristics of mature mRNA and rRNA genes from *D. melanogaster* mitochondria showed that UCG serves as the start codon of its *cox1* gene [20]. The result is consistent with predictions of the start of *cox1* suggested by comparison of conserved amino acid positions [21]. By comparing amino acid sequences of *cox1* in all sequenced Orthoptera, we observed that the first conserved codon is the TCN serine codon, downstream of which at least three codon positions are also conserved and there are no standard ATN bases (Additional file 2). Thus, in both *Sinochlora* species, the conserved TCT serine codon may also serve as a start codon. Concerning the *nad6* gene, it has been proposed to start with ATN codons in other sequenced orthopterans; however, there are no conserved amino acid positions within the span of over 50 amino acids downstream of *trnP* (Additional file 3). Then we propose that the *nad6* may start with CTG in *S. longifissa* and TTG in *S. retrolateralis*, considering previous designation of such start codons [22,23]. The two start codons, creating no IGS or overlap between *trnP* and *nad6* genes, also appear to be more plausible in the evolutionary economic perspective [22].

Two standard stop codons TAA/TAG and two incomplete stop codons T/TA are utilized in the PCGs. For *S. longifissa*, six PCGs (*nad2*, *atp8*, *atp6*, *cox3*, *nad3*, and *nad4L*) terminate with TAA, one (*nad1*) with TAG, one (*nad6*) with TA, and the other four (*cox1*, *nad5*, *nad4*, and *cob*) with T. The stop codons differ between the two congeners in that TAA is utilized as the *cox2* stop codon, and TA is utilized as the *atp6* and *cox3* stop codon in *S. retrolateralis*. A partial T or TA stop codon, which was proposed to create complete TAA stop codons via posttranscriptional polyadenylation [24], is also present in other metazoan mitochondrial genes.

### Novel gene order rearrangement involving the control region

One of our most significant findings is the novel gene order rearrangement "*rrnS*-*trnI*-*trnM*-*nad2*-CR-*trnQ*-*trnW*" in both *Sinochlora* species (Figure 1). The two mitogenomes are the first representatives that have a large-scale translocation involving the CR in Orthoptera. Such gene order has not been observed in other sequenced insect mitogenomes. The gene cluster *rrnS*-CR-*trnI-trnQ-trnM-nad2* in the proposed ancestral mitogenome has been discovered to function as a hot spot for gene rearrangements in arthropod mitogenomes. Duplicate *trnI* and partial *trnQ* genes have been discovered in the CR close to the *rrnS* gene in some blowflies [25,26]. In a mantid, a complicate set of repeat units dispersing in both ends of the CR also translocated between *trnM* and *nad2*[27]. A plague thrips displayed CR duplications that are distant from the rRNA genes [28]. Various locations of the CR and neighboring tRNA genes have been exhibited in lice [29]. Location variability of the putative CR has also been reported as a product of the tRNA gene translocation, such as *rrnS-trnQ*-CR-*trnI-trnS(UCN)-trnM-nad2* in a springtail [30], or the tRNA and rRNA gene translocation, such as *rrnL-trnV-trnS(UCN)*-*trnC*-CR-*rrnS* in a mite [31]. The CR and the neighboring genes have also been found to be duplicated in some ticks [32].

### Organization of the control region

**Figure 3:**
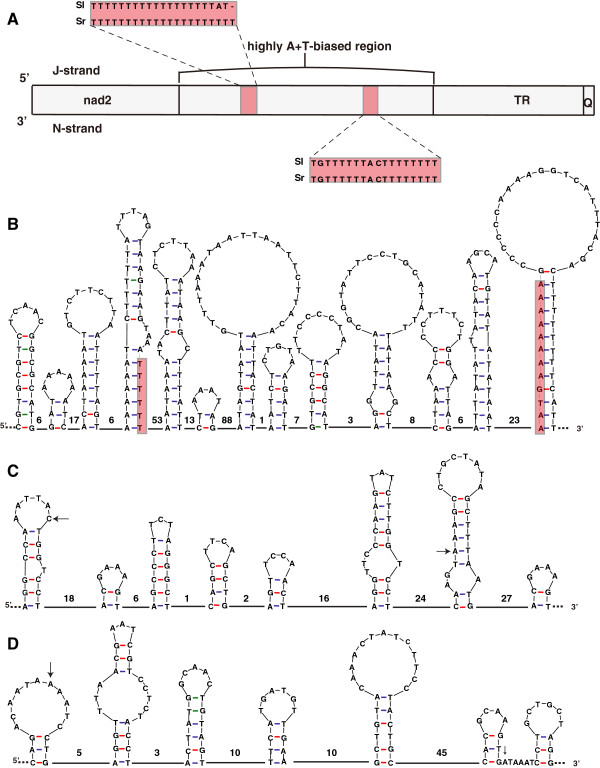
**Organization of the control region.** (**A**) Structure of the control region and its neighbourhood. (**B**) Shared potential stem-loop structures situated in the highly A+T-biased region on the J-strand (depicted as in *S. longifissa*). Predicted secondary structures of the first tandem repeat and flanking junctions in *S. longifissa* (**C**) and in *S. retrolateralis* (**D**). *Sl*, *S. longifissa*; *Sr*, *S. retrolateralis*. T-stretch is highlighted in carmine. Arrows indicate the initiation location of a certain repeat unit. The numbers of bases between hairpins are shown above the line. Gene lengths are not to scale.

For both *Sinochlora* species, the putative CR is composed of two major sections with different nucleotide compositions: a highly A+T-biased region (ATR) adjacent to *nad2* and several GC-rich TR units adjacent to *trnQ*. For the former, the A+T content is 75.70% in *S. longifissa* and 75.61% in *S. retrolateralis*. By contrast, the TR region has low bias toward A+T (55.52% in *S. longifissa* and 58.26% in *S. retrolateralis*). The ATR is conserved between the two congeners in length, nucleotide composition and secondary structure. Its length is 1,391 bp in *S. longifissa* and 1,386 bp in *S. retrolateralis*, consistent with the A+T-rich region of other orthopterans [4,33]. The two species share 82.6% sequence identity in this region. A long T-stretch and several conserved potential hairpin structures are thought to play significant roles in initiating and/or regulating the transcription and replication of mtDNA [8]. They were also found in the both *Sinochlora* species. The long T-stretch (> 18 bp) is situated in the initial quarter of the ATR on the J-strand, and a similar T-stretch (> 8 bp) is situated in the initial third of the ATR on the minority strand (N-strand) (Figure 3A). The two species also share 97.10% sequence identity in the domain located between these two T-stretches. The domain has seven similar sites capable of forming 8- to 33-bp stem-loop structures (Figure 3B). Additionally, there are three similar sites for the formation of 11- to 25-bp stable hairpins, with 7- to 11-bp loops supported by 1–5 GC matches in the stems, adjacent to the 5’ end of the T-stretch on the J-strand (Figure 3B).

In *S. longifissa*, the TR region is 1,731 bp in length, comprising nine full 175-bp copies and a partial 153-bp copy. In *S. retrolateralis*, a total of five 175-bp repeat copies have been successfully sequenced from both ends of the TR; however, we failed to sequence through the whole TR region due to the presence of a large number of repeat copies. We estimate that there are twelve 175-bp tandem copies in *S. retrolateralis* based on the length (~2,100 bp) of the TR indicated by gel electrophoresis. The consensus TR motifs of the two species share 61.2% sequence identity. In *S. longifissa*, four of the nine TR motifs are identical to the consensus TR motif, and the other five have a few mutations and/or deletions. Among the nine TR motifs, all but the fourth possess an ORF. The consensus ORF could be translated into 58 amino acids, starting with lysine (AAA) and ending with valine (GTG) (Additional file 4). Low shared identities (< 45%) with nuclear sequences in search of the GenBank database exclude the possibility that the ORF is transferred from the nuclear genome. It has 47.1% identity with the antisense strand of *nad4*, and 45.9% identity with the sense strand of *cox1*. The shared bases are scattered in alignment, among which over 60% are A and T (Additional file 5). This suggests that the ORF might not be obtained through horizontal transfer between mitochondria. In contrast, no ORF could be found in the *S. retrolateralis* TR motif.

The TR motif could be duplicated through slipped-strand mispairing [34,35]. Moreover, potential stem-loop structures in a repeated unit and its flanking part have been demonstrated to cause an increase in slipped-strand mispairing frequency [35,36]. Stem-loop structures were detected in the TR region of the two *Sinochlora* species. For example, in the first TR unit and its junctions at both ends in *S. longifissa*, the nucleotide sequence can potentially form seven 8- to 27-bp hairpins with 5- to 10-bp loops supported by 1–5 GC matches in the stem (Figure 3C). In the corresponding regions in *S. retrolateralis*, the nucleotide sequence can potentially form six 10- to 29-bp hairpins with 5- to 15-bp loops supported by 1–4 GC matches in the stem (Figure 3D). Similar complicated hairpin structures in TR were also detected in the louse *Bothriometopus*[23] and termites [37,38]. However, there are no conserved stem-loops among the TRs and at the joints in these insects.

### Unassigned intergenic spacers and *trnS(UCN)* pseudogenes

Eleven IGSs with identical locations are present in *S. longifissa* (totalling 318 bp) and *S. retrolateralis* (263 bp); one additional IGS is situated between *atp6* and *cox3* in *S. longfissa* (2 bp) and between *cox2* and *trnK* in *S. retrolateralis* (1 bp) (Table 1). The largest IGS (161 bp for *S. longifissa* and 94 bp for *S. retrolateralis*) lies between *trnS(UCN)* and *nad1*. By comparing the IGS of currently available Orthoptera mitogenomes, we found a 7-bp conserved motif (THYTHDA) downstream the *nad1* across Orthoptera, with the only exception of *Mekongiella xizangensis* (Additional file 6). The conserved motif has been proposed as a binding site for mitochondrial transcription termination factor (mtTERM) in Orthoptera [4]. Similar conserved motifs, which were proposed as a binding site of the mtTERM, between *trnS(UCN)* and *nad1* have also been found in Lepidoptera [39], and Coleoptera [40]. It has been confirmed that one of the two binding sites of mtTERM lies downstream of *nad1* in *Drosophila*[41,42].

**Figure 4:**
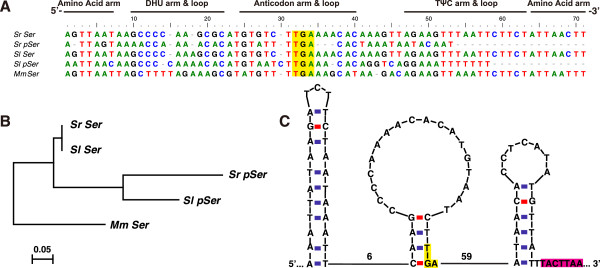
**Analysis of the *trnS(UCN) *pseudogenes.** (**A**) Alignment of the *trnS(UCN)* gene and its pseudogenes in the two *Sinochlora* species; (**B**) Minimum Evolution phylogram inferred from the alignment; (**C**) secondary structures in the IGS between *trnS(UCN)* and *nad1* in *S. longifissa*. The nucleotides highlighted in yellow and carmine represent the anti-codon and the conserved motif related to mtTERM, separately. The number of bases between hairpins are shown in figure **C**. *Sr*, *S. retrolateralis*; *Sl*, *S. longifissa*; *Mm*, *Myrmecophilus manni*; *Ser*, *trnS(UCN)*; *pSer*, *trnS(UCN)* pseudogenes.

In the IGS region between *trnS(UCN)* and *nad1*, we detected the vestige of one additional *trnS(UCN)* gene copy in both *Sinochlora* species. While both *trnS(UCN)* pseudogenes possess the same anticodon to the functional *trnS(UCN)*, they are unable to form stable cloverleaf secondary structures (Figure 4). Relative-rate tests show that both pseudogenes evolved more than twice as fast as their functional paralogs (Table 2). This suggests that the redundant *trnS(UCN)* pseudogenes have experienced more relaxed selective constraints. In addition, the IGS region in *S. longifissa* could be folded into three 19- to 25-bp stable stem-loop structures (Figure 4C). The anticodon lies in the stem of the second hairpin and the 7-bp conserved motif aforementioned lies adjacent to the 3’ end of the third hairpin (Figure 4C). In contrast, there is no hairpin in the IGS region in *S. retrolateralis*.

**Table 2:** **Relative-Rate Test results for contrasts between*trnS(UCN)*genes and pseudogenes**

**Contrast**	**Rates**	**SD**	**P Value**
All *S.* Ser versus all *S.* pSer	0.338989 versus 0.675022	0.1326	0.0113*
*Sr-*Ser vesus *Sr*-pSer	0.364826 versus 0.791345	0.1855	0.0215*
*Sl-*Ser vesus *Sl*-pSer	0.297057 versus 0.604668	0.1299	0.0179*

Results of all possible pairwise contrasts between *trnS(UCN)* genes and *trnS(UCN)* pseudogenes are significant (P < 0.01). SD, standard deviation; *Sr*, *S. retrolateralis*; *Sl*, *S. longifissa*; *Mm*, *Myrmecophilus manni*; *Ser*, *trnS(UCN)*; *pSer*, *trnS(UCN)* pseudogenes.

### Mechanism of genome rearrangements

**Figure 5:**
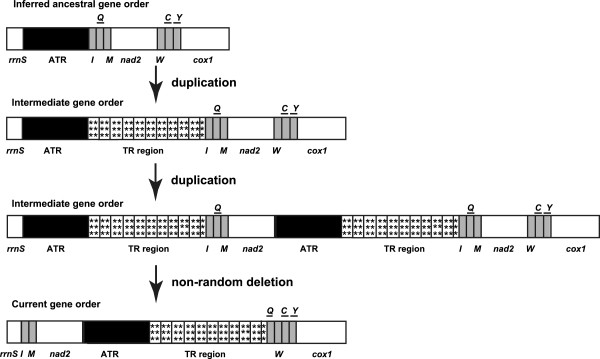
**Hypothesized duplication/non-random loss model for derived gene order in the two *Sinochlora *species.** Single letters refer to tRNA genes for the corresponding amino acid. Genes are transcribed from left to right, except those underlined to indicate an opposite direction. Gene lengths are not to scale.

The predominant mechanism of mitogenome rearrangements is considered to be partial genome duplication followed by a random [34,43,44] or non-random loss of the duplicated gene copies [45]. For the rearrangement involving the CR neighbourhood, we propose that the ancestor of the two *Sinochlora* species possessed the same gene order as ancestral insects [1]. Firstly, the gene cluster CR-*trnI*-*trnQ*-*trnM*-*nad2* was duplicated, likely promoted by the stem-loop structures detected in the CR (Figure 3C,D). Subsequently, non-random deletions might happen to the entire subset of the duplicated genes, dependent on the gene copy’s transcriptional polarity and location in the genome, because the CR includes a transcriptional control sequence [45]. Namely, the redundant genes which possess the same polarity, such as the redundant gene cluster *trnI*-*trnM*-*nad2*, were successively deleted during replication (Figure 5). By contrast, persistence of the *trnS(UCN)* pseudogenes may have resulted from the tandem duplication of *trnS(UCN)* followed by a subsequent random loss of the *trnS(UCN)* paralogs. Apparently, more knowledge of transcription signals in insect mitogenomes and sampling additional closely related taxa will be helpful to understand the rearrangement mechanism.

### Recognition sequences of replication origins

**Figure 6:**
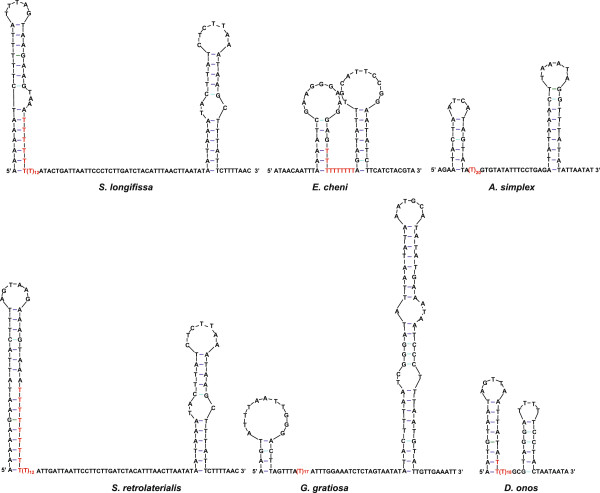
**Potential stem-loop structures adjacent to T-stretches in the superfamily Tettigonioidea.** The nucleotides highlighted in red represent the location of the T-stretch.

**Figure 7:**
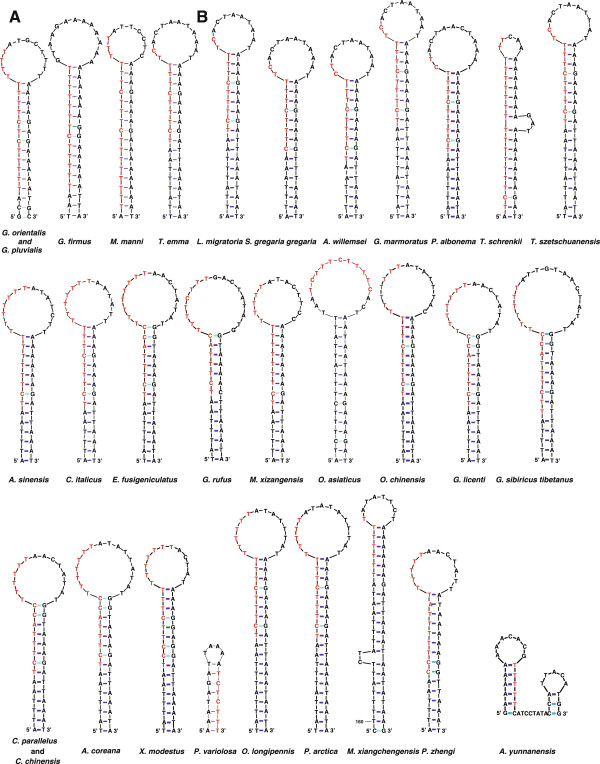
**Potential stem-loop structures including T-stretch variants immediately upstream of O**_**N**_**.** The structure in the superfamily Grylloidea (**A**) and in the suborder Caelifera (**B**). T-stretch variants are highlighted in red.

The T-stretch is involved in the recognition of the O_R_ of mtDNA at least among holometabolous insects, whereas similar T-stretch was not found in the upstream portion of the O_R_ for *L. migratoria* (hemimetabolous insect) [8]. However, after a thorough re-investigation, we detected long T-stretches or variants of T-stretch in the putative CR of available Orthoptera mitogenomes (Additional file 7). The long T-stretch can be observed in the available sequences of all katydids (Figure 6), except *Ruspolia dubia*, and varies in size from 10 (*E. cheni*) to 21 bp (*S. retrolateralis*). The T-stretches could participate in the formation of or be positioned adjacent to a possible stem-loop structure. By contrast, in crickets and grasshoppers, T-stretch variants can be detected within a potential stem-loop structure (Figure 7). In the T-stretch variants, a few transitions between thymine and cytosine occurred to form (T)nC(T)n sequences. The T-stretch variants lie proximal to the middle part of the CR in the crickets, varying in size from 13 to 19 bp (Figure 7A). The T-stretch variants vary in size from 16 to 18 bp in grasshoppers (Figure 7B), with the exception of *Alulatettix yunnanensis*. In *A. yunnanensis*, a small T-stretch could be observed in the stem portion of a hairpin structure, similar to that observed in katydids (Figure 7B).

Human mtDNA synthesis is initiated from the sites near the stem base of the secondary structure located around the light strand origin, and the small T-stretch (6–11 bp) located in the loop portion participates in the initiation process [46]. The secondary structure in human and other vertebrate mtDNA is very similar to the aforementioned stem-loop structure in the orthopterans [8]. Therefore, the long T-stretch, which participates in the formation of or is adjacent to a possible stem-loop structure in katydids, and the T-stretch variants within the stem-loop structure of crickets and most grasshoppers, could also play a crucial role in mtDNA replication initiation. Notably, the T-stretch in cockroaches and termites also participates in the formation of a certain stem-loop structure (Additional file 8). However, the stem-loop structure around the mtDNA O_R_ could not be detected in *Drosophila*[8]. Therefore, the O_R_ recognition sequences of mtDNA, although generally detected in Orthoptera, have diverged not only among Orthoptera but also throughout insect evolution.

## Conclusions

The two *Sinochlora* congeners represent the first two orthopterans that have a large-scale translocation involving the CR. It seems that the present mitogenome rearrangements are a consequence of tandem duplication followed by non-random loss of paralogs. However, future research including additional taxon sampling is needed to determine rearrangement mechanisms and evolutionary processes. Comparison of the O_R_ recognition sequences among Orthoptera and other insects will aid in further understanding of mechanisms underlying mtDNA replication. Divergence in nucleotide bias and skew of mtDNA exists between the two suborders of Orthoptera. Future studies on mtDNA-based phylogeny of Orthoptera should therefore take into consideration the base compositional heterogeneity, which could lead to incorrect phylogenetic inferences [47,48].

## Methods

### Taxon sampling and mitochondria DNA extraction

Specimens of *S. longifissa* and *S. retrolateralis* were collected from Wuyi Mountain, Fujian, South China in 2005. The specimens were preserved in 95% ethanol and stored at 4°C. The mitochondria were isolated as previously described [49], and mtDNA was extracted with the DNeasy Blood & Tissue Kit (Qiagen).

### Genome determination

First, short gene regions within individual genes (*cox1*, *cox3*, *cytb*, *nad1*, *rrnL*, and *rrnS*) were amplified and sequenced using listed primers (Additional file 9). Then the obtained sequences were used to design specific primers for amplifying overlapping fragments spanning the whole mitogenomes.

Fragments larger than 3 kb were amplified using TaKaRa LA Taq™ (Takara, Dalian, China), with the following cycling conditions: an initial denaturation at 94°C for 3 min, followed by 36 cycles of denaturation at 94°C for 30 s, annealing at 50–57°C for 30 s, and extension at 68°C for 3–8 min (1 kb/min), with a final elongation at 68°C for 6 min after the last cycle. 16 fragments smaller than 3 kb were performed using TaKaRa ExTaq™ or TaKaRa rTaq™ (Takara, Dalian, China), with the following cycling conditions: an initial denaturation at 94°C for 3 min, followed by 36 cycles of denaturation at 94°C for 30 s, annealing at 45–60°C for 30 s, and extension at 72°C for 1–2 min (1 kb/min), with a final elongation at 72°C for 6 min after the last cycle. After purification with AxyPrep™ DNA Gel Extraction Kit, most PCR products were directly sequenced by means of primer walking, and other fragments were cloned into the pGEM-T Easy vector (Promega, USA) prior to sequencing.

Concerning the CR, long PCR amplicons were successfully amplified, which encompassed the entire CR from *nad1* to *cox2* genes for both species, but gel electrophoresis showed multiple bands. The largest and brightest band was chosen to clone into the pGEM-T Easy vector for sequencing. Sequencing primers were designed from flanking regions of the whole TR region and subsequently a 400-bp sequence at each end was obtained. The obtained TR units were analyzed with the TRF4.0 software [50] in order to design suitable primers for walking. For *S. longifissa*, the complete TR region was sequenced using specific primers that was designed based on a mutant poly C (8 continuous C) (Additional file 4) in one of the TR units. For *S. retrolateralis*, the length of the PCR product indicates that the TR region is about 2,100 bp, suggesting that there are 12 complete tandem repeats; however, only 5 of these tandem repeats were sequenced.

### Sequence assembly, annotation and secondary structure prediction

The complete mitogenome sequences were assembled using the SeqMan program from the Lasergene package software (DNAStar, Madison, WI). tRNA genes were identified by their cloverleaf secondary structures using tRNAscan-SE 1.21 [51]. The locations of 13 PCGs and rRNA genes were determined by comparison of homologous sequences with other sequenced orthopterans using the CLUSTAL W programs [52]. Nucleotide composition statistics, nucleotide bias and skew of the orthopterans (Additional file 10) except those at P_4fd_, were retrieved from the METAMiGA database [53]. Skewness was calculated to describe strand bias [54], which measures the relative number of As to Ts (AT skew = [A - T]/[A + T]) and Gs to Cs (GC skew = [G - C]/[G + C]). We obtained codon usage from the SMS2 website [55] and computed AT and GC skew at the P_4fd_ on the J-strand. Potential stem-loop structures of the polyneopterans (Additional file 10) were predicted by the Mfold software [56]. Repeat sequences were identified with the TRF4.0 software. ORF was detected using the MEGA5 software [57].

### Phylogenetic analysis

Divergence and substitution rates between *trnS(UCN)* genes and pseudogenes were investigated, using *trnS(UCN)* of *Myrmecophilus manni*[33] as outgroup. Gapped positions were eliminated from the resulted alignment. The remaining 44 sites (including 26 parsimony informative) were used to reconstruct a distance phylogeny by Minimum Evolution using JC distances [58], due to the low number of sites analyzed. Relative-rate tests [59] were used to calculate substitution rates employing RRTree version 1.1.11 [60], presuming JC distances.

## Abbreviations

atp6 and atp8: ATP synthase subunits 6 and 8;cob: Cytochrome b;cox1-3: Cytochrome c oxidase subunits 1 to 3;nad1–6 and nad4L: NADH dehydrogenase subunits 1 to 6 and 4L;rrnS and rrnL: Small and large ribosomal RNA (rRNA) subunits;trnX: Transfer RNA genes where X is the one-letter abbreviation of the corresponding amino acid

## Competing interests

The authors declare that they have no competing interests.

## Authors' contributions

CL was responsible for the design, coordination, and conduction of the research, carried out the experimental design, and drafted the manuscript, tables, and figures. JC determined and assembled the mtDNA sequences. CM, LL, and SYZ participated in genomic analysis. CM extensively revised the manuscript. All authors read and approved the final manuscript.

## Supplementary Material

Additional file 1 **Nucleotide composition of functional regions in the mitogenomes. ***Sl, S. longifissa; Sr, S. retrolateralis*. Click here for file

Additional file 2 **Initiation codons of *cox1 *in Orthoptera.** The nucleotides highlighted in gray represent the location of *trnY*. The bases in box indicate proposed initiation codons of *cox1*. Click here for file

Additional file 3 **Initiation codons of *nad6 *in Orthoptera.** The nucleotides highlighted in gray represent the location of *trnP*. The bases in box indicate proposed initiation codons of *nad6*. Click here for file

Additional file 4 **Alignment of sequences of the TR motifs between the two *Sinochlora *species.** (A) Alignment of the nucleotide sequences; (B) Alignment of the amino acid sequences. *Sl*, *S. longifissa*; *Sr*, *S. retrolateralis*; repX: tandem repeat motifs, where X is the ordinal number. Dashes indicate alignment gaps. Dots indicate nucleotides (A) or amino acids (B) that are the same as the first repeat motif of *S. retrolateralis*. Asterisks (B) indicate stop codons. Poly C sites are shaded. Click here for file

Additional file 5 **Alignment of the ORF sequences with *cox1 *(A) and *nad4 *(B) in *Sinochlora longifissa. *** Both *cox1* and *nad4* sequences are from the J-strand. Conserved A and T bases are highlighted in yellow boxes, whereas conserved G and C bases are in blue. Click here for file

Additional file 6 **Alignment of the non-coding spacer between *trnS(UCN) *and *nad1.*** The 7-bp conserved motif (THYTHDA) across Orthoptera is boxed. However, the motif is not present in *Mekongiella xizangensis*. Click here for file

Additional file 7 **Comparison among the sequences in the control region of the template strands in Orthoptera.** The portion is next to the *rrnS* gene except that in the two *Sinochlora* species the sequences are the portion that is next to *nad2*. Location of the free 5’ ends marking the O_N_ of *L. migratoria* [4] is indicated. Large arrowheads indicate the sites where major signals were observed and small arrowheads show the sites where minor signals were observed. Arrows indicate the direction of replication. The nucleotide sequence of *L. migratoria*, which potentially forms the stem-loop structure upstream of the O_N_, is underlined. The nucleotides highlighted in red represent the location of T-stretch or T-stretch variant.) Click here for file

Additional file 8 **The potential stem-loop structure involving a T-stretch or T-stretch variant in cockroaches (A) and termites (B).** The nucleotides highlighted in green represent the T-stretch variant. Click here for file

Additional file 9 PCR primers used in the present study. Click here for file

Additional file 10 List of taxa used in the present analysis. Click here for file
