# Supplementary material for: Mitochondrial genomes of two Sinochlora species (Orthoptera): novel genome rearrangements and recognition sequence of replication origin
Source: BMC Genomics. 2013 Feb 20;14:114. doi: 10.1186/1471-2164-14-114 (PMC3630010; doi:10.1186/1471-2164-14-114)
Supplement: Additional file 2 — Initiation codons of cox1 in Orthoptera. The nucleotides highlighted in gray represent the location of trnY. The bases in box indicate proposed initiation codons of cox1. [file 1471-2164-14-114-S2.pdf]

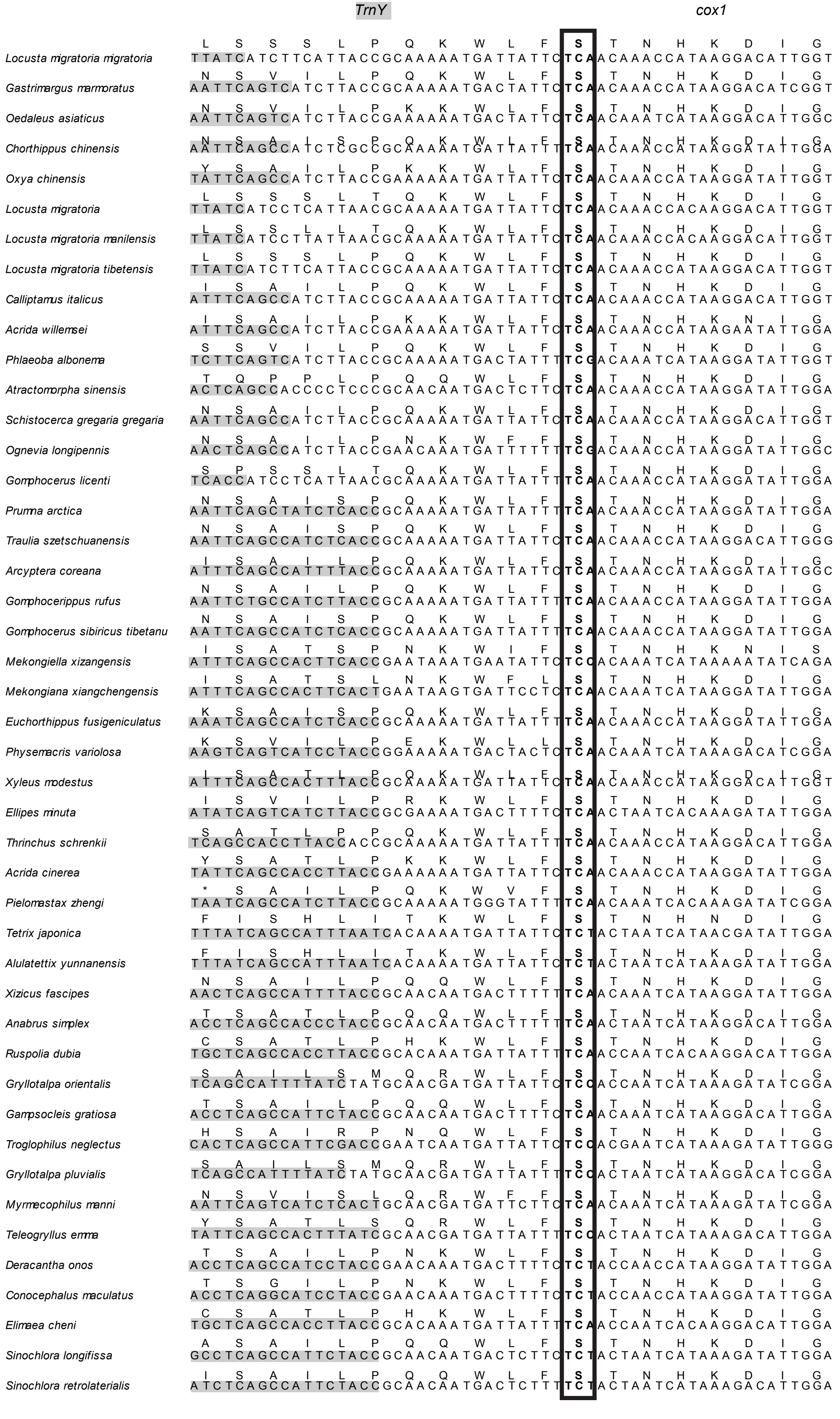
TrnY
cox1
Locusta migratoria migratoria
Gastrimargus marmoratus
Oedaleus asiaticus
Chorthippus chinensis
Oxya chinensis
Locusta migratoria
Locusta migratoria manilensis
Locusta migratoria tibetensis
Calliptamus italicus
Acrida willemsei
Phlaeoba albonema
Atractomorpha sinensis
Schistocerca gregaria gregaria
Ognevia longipennis
Gomphocerus licenti
Prumna arctica
Traulia szetschuanensis
Arcyptera coreana
Gomphocerippus rufus
Gomphocerus sibiricus tibetanu
Mekongiella xizangensis
Mekongiana xiangchengensis
Euchorthippus fusigeniculatus
Physemacris variolosa
Xyleus modestus
Ellipes minuta
Thrinchus schrenkii
Acrida cinerea
Pielomastax zhengi
Tetrix japonica
Alulatettix yunnanensis
Xizicus fascipes
Anabrus simplex
Ruspolia dubia
Gryllotalpa orientalis
Gampsocleis gratiosa
Troglophilus neglectus
Gryllotalpa pluvialis
Myrmecophilus manni
Teleogryllus emma
Deracantha onos
Conocephalus maculatus
Elimaea cheni
Sinochlora longifissa
Sinochlora retrolaterialis
