# Supplementary material for: Mitochondrial genomes of two Sinochlora species (Orthoptera): novel genome rearrangements and recognition sequence of replication origin
Source: BMC Genomics. 2013 Feb 20;14:114. doi: 10.1186/1471-2164-14-114 (PMC3630010; doi:10.1186/1471-2164-14-114)
Supplement: Additional file 3 — Initiation codons of nad6 in Orthoptera. The nucleotides highlighted in gray represent the location of trnP. The bases in box indicate proposed initiation codons of nad6. [file 1471-2164-14-114-S3.pdf]

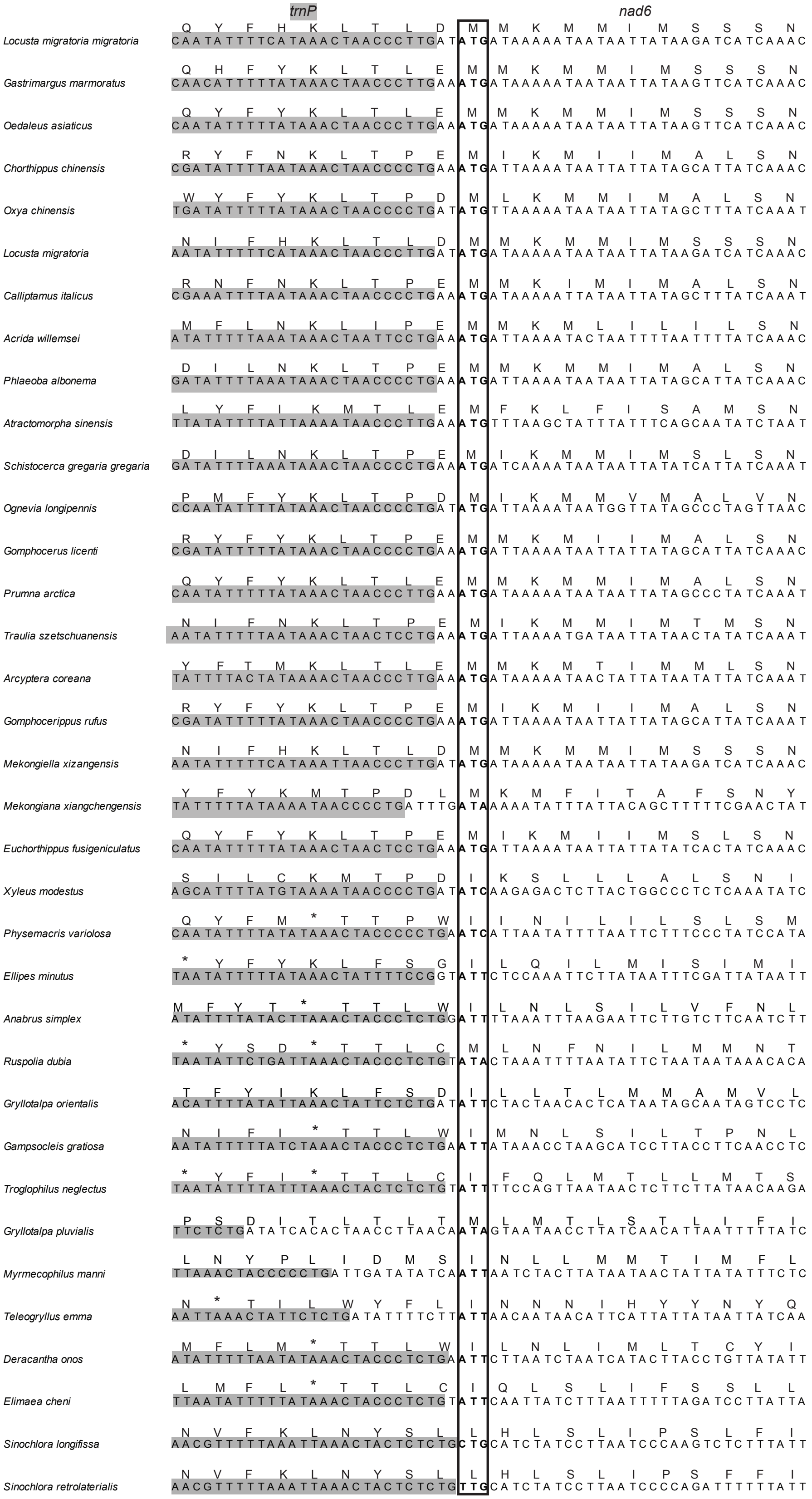
trnP
nad6
Locusta migratoria migratoria
Q Y F H K L T L D M M K M M I M S S S N
CAATATTTTCATAAACTAACCCTTGATATGATAAAAATAATAATTATAAGATCATCAAAC
Gastrimargus marmoratus
Q H F Y K L T L E M M K M M I M S S S N
CAACATTTTTATAAACTAACCCTTGAAATGATAAAAATAATAATTATAAGTTCATCAAAC
Oedaleus asiaticus
Q Y F Y K L T L E M M K M M I M S S S N
CAATATTTTTATAAACTAACCCTTGAAATGATAAAAATAATAATTATAAGTTCATCAAAC
Chorthippus chinensis
R Y F N K L T P E M I K M I I M A L S N
CGATATTTTAATAAACTAACCCCTGAAATGATTAAAATAATTATTATAGCATTATCAAAC
Oxya chinensis
W Y F Y K L T P D M L K M M I M A L S N
TGATATTTTTATAAACTAACCCCTGATATGTTAAAAATAATAATTATAGCTTTATCAAAT
Locusta migratoria
N I F H K L T L D M M K M M I M S S S N
AATATTTTTCATAAACTAACCCTTGATATGATAAAAATAATAATTATAAGATCATCAAAC
Calliptamus italicus
R N F N K L T P E M M K I M I M A L S N
CGAAATTTTAATAAACTAACCCCTGAAATGATAAAAATTATAATTATAGCTTTATCAAAT
Acrida willemsei
M F L N K L I P E M M K M L I L I L S N
ATATTTTTAAATAAACTAATTCCTGAAATGATTAAAATACTAATTTTAATTTTATCAAAC
Phlaeoba albonema
D I L N K L T P E M M K M M I M A L S N
GATATTTTAAATAAACTAACCCCTGAAATGATTAAAATAATAATTATAGCATTATCAAAC
Atractomorpha sinensis
L Y F I K M T L E M F K L F I S A M S N
TTATATTTTATTAAAATAACCCTTGAAATGTTTAAGCTATTTATTTCAGCAATATCTAAT
Schistocerca gregaria gregaria
D I L N K L T P E M I K M M I M S L S N
GATATTTTAAATAAACTAACCCCTGAAATGATCAAAATAATAATTATATCATTATCAAAT
Ognevia longipennis
P M F Y K L T P D M I K M M V M A L V N
CCAATATTTTATAAACTAACCCCTGATATGATTAAAATAATGGTTATAGCCCTAGTTAAC
Gomphocerus licenti
R Y F Y K L T P E M M K M I I M A L S N
CGATATTTTTATAAACTAACCCCTGAAATGATTAAAATAATTATTATAGCATTATCAAAC
Prumna arctica
Q Y F Y K L T L E M M K M M I M A L S N
CAATATTTTTATAAACTAACCCTTGAAATGATAAAAATAATAATTATAGCCCTATCAAAT
Traulia szetschuanensis
N I F N K L T P E M I K M M I M T M S N
AATATTTTTAATAAACTAACTCCTGAAATGATTAAAATGATAATTATAACTATATCAAAT
Arcyptera coreana
Y F T M K L T L E M M K M T I M M L S N
TATTTTACTATAAAACTAACCCTTGAAATGATAAAAATAACTATTATAATATTATCAAAT
Gomphocerippus rufus
R Y F Y K L T P E M I K M I I M A L S N
CGATATTTTTATAAACTAACCCCTGAAATGATTAAAATAATTATTATAGCATTATCAAAT
Mekongiella xizangensis
N I F H K L T L D M M K M M I M S S S N
AATATTTTTCATAAATTAACCCTTGATATGATAAAAATAATAATTATAAGATCATCAAAC
Mekongiana xiangchengensis
Y F Y K M T P D L M K M F I T A F S N Y
TATTTTTATAAAATAACCCCTGATTTGATAAAAATATTTATTACAGCTTTTTCGAACTAT
Euchorthippus fusigeniculatus
Q Y F Y K L T P E M I K M I I M S L S N
CAATATTTTTATAAACTAACTCCTGAAATGATAAAAATAATTATTATATCACTATCAAAC
Xyleus modestus
S I L C K M T P D I K S L L L A L S N I
AGCATTTTATGTAAAATAACCCCTGATATCAAGAGACTCTTACTGGCCCTCTCAAATATC
Physemacris variolosa
Q Y F M * T T P W I I N I L I L S L S M
CAATATTTTATATAAACTACCCCCTGAATCATTAATATTTTAATTCTTTCCCTATCCATA
Ellipes minutus
* Y F Y K L F S G I L Q I L M I S I M I
TAATATTTTTATAAACTATTTTCCGGTATTCTCCAAATTCTTATAATTTCGATTATAATT
Anabrus simplex
M F Y T * T T L W I L N L S I L V F N L
ATATTTTATACTTAAACTACCCTCTGGATTTTAAATTTAAGAATTCTTGTCTTCAATCTT
Ruspolia dubia
* Y S D * T T L C M L N F N I L M M N T
TAATATTCTGATTAAACTACCCTCTGTATACTAAATTTTAATATTCTAATAATAAACACA
Gryllotalpa orientalis
T F Y I K L F S D I L L T L M M A M V L
ACATTTTATATTAAACTATTCTCTGATATTCTACTAACACTCATAATAGCAATAGTCCTC
Gampsocleis gratiosa
N I F I * T T L W I M N L S I L T P N L
AATATTTTTATCTAAACTACCCTCTGAATTATAAACCTAAGCATCCTTACCTTCAACCTC
Troglophilus neglectus
* Y F I * T T L C I F Q L M T L L M T S
TAATATTTTATTTAAACTACTCTCTGTATTTTCCAGTTAATAACTCTTCTTATAACAAGA
Gryllotalpa pluvialis
P S D I T L T L T M L M T L S T L I F I
TTCTCTGATATCACACTAACCTTAACAATAGTAATAACCTTATCAACATTAATTTTTATC
Myrmecophilus manni
L N Y P L I D M S I N L L M M T I M F L
TTAAACTACCCCCTGATTGATATATCAATTAATCTACTTATAATAACTATTATATTTCTC
Teleogryllus emma
N * T I L W Y F L I N N N I H Y Y N Y Q
AATTAAACTATTCTCTGATATTTTCTTATTAACAATAACATTCATTATTATAATTATCAA
Deracantha onos
M F L M * T T L W I L N L I M L T C Y I
ATATTTTTAATATAAACTACCCTCTGAATTCTTAATCTAATCATACTTACCTGTTATATT
Elimaea cheni
L M F L * T T L C I Q L S L I F S S L L
TTAATATTTTTATAAACTACCCTCTGTATTCAATTATCTTTAATTTTTAGATCCTTATTA
Sinochlora longifissa
N V F K L N Y S L L H L S L I P S L F I
AACGTTTTTAAATTAAACTACTCTCTGCTGCATCTATCCTTAATCCCAAGTCTCTTTATT
Sinochlora retrolateralis
N V F K L N Y S L L H L S F F I
AACGTTTTTAAATTAAACTACTCTCTGTTGCATCTATCCTTAATCCCCAGATTTTTTATT
