# Supplementary material for: Mitochondrial genomes of two Sinochlora species (Orthoptera): novel genome rearrangements and recognition sequence of replication origin
Source: BMC Genomics. 2013 Feb 20;14:114. doi: 10.1186/1471-2164-14-114 (PMC3630010; doi:10.1186/1471-2164-14-114)
Supplement: Additional file 4 — Alignment of sequences of the TR motifs between the two Sinochlora species. (A) Alignment of the nucleotide sequences; (B) Alignment of the amino acid sequences. Sl, S. longifissa; Sr, S. retrolateralis; repX: tandem repeat motifs, where X is the ordinal number. Dashes indicate alignment gaps. Dots indicate nucleotides (A) or amino acids (B) that are the same as the first repeat motif of S. retrolateralis. Asterisks (B) indicate stop codons. Poly C sites are shaded. [file 1471-2164-14-114-S4.pdf]

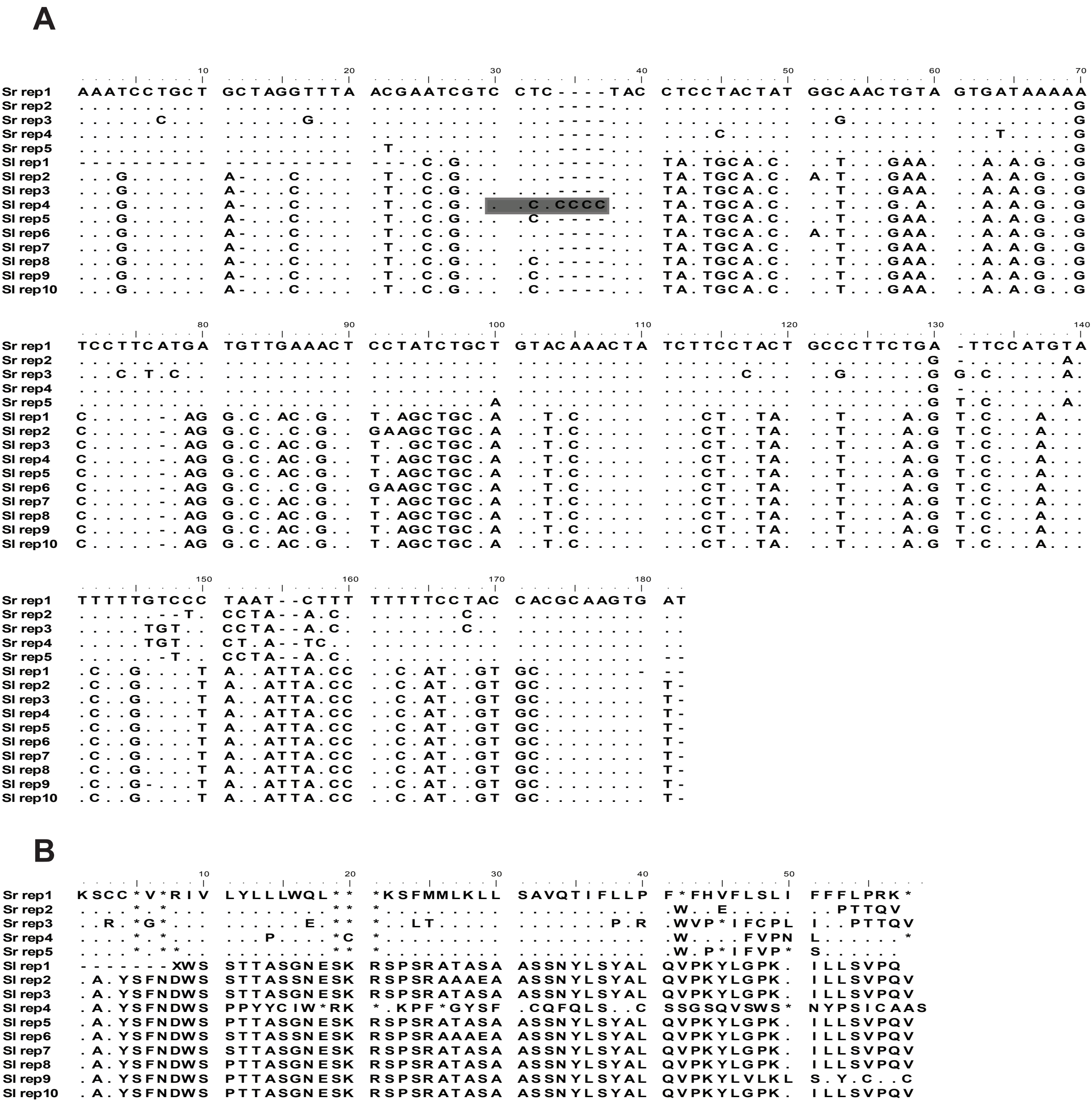
A
10 20 30 40 50 60 70
Sr rep1 AAATCCTGCT GCTAGGTTTA ACGAATCGTC CTC----TAC CTCCTACTAT GGCAACTGTA GTGATAAAAA
Sr rep2 ....----... ....G
Sr rep3 ......C... ......G... ....----... ....G....... ....G
Sr rep4 ....----... ....C..... ....T.....G
Sr rep5 .... .T........ ...----... ....G
Sl rep1 ---------- ---------- ----C.G... ...----... TA.TGCA.C. ..T...GAA. ..A.A.G..G
Sl rep2 ...G...... A-...C.... .T..C.G... ...----... TA.TGCA.C. A.T...GAA. ..A.A.G..G
Sl rep3 ...G...... A-...C.... .T..C.G... ...----... TA.TGCA.C. ..T...GAA. ..A.A.G..G
Sl rep4 ...G...... A-...C.... .T..C.G... .C.CCCC... TA.TGCA.C. ..T...G.A. ..A.A.G..G
Sl rep5 ...G...... A-...C.... .T..C.G... .C.----... TA.TGCA.C. ..T...GAA. ..A.A.G..G
Sl rep6 ...G...... A-...C.... .T..C.G... ...----... TA.TGCA.C. A.T...GAA. ..A.A.G..G
Sl rep7 ...G...... A-...C.... .T..C.G... ...----... TA.TGCA.C. ..T...GAA. ..A.A.G..G
Sl rep8 ...G...... A-...C.... .T..C.G... .C.----... TA.TGCA.C. ..T...GAA. ..A.A.G..G
Sl rep9 ...G...... A-...C.... .T..C.G... .C.----... TA.TGCA.C. ..T...GAA. ..A.A.G..G
Sl rep10 ...G...... A-...C.... .T..C.G... .C.----... TA.TGCA.C. ..T...GAA. ..A.A.G..G
80 90 100 110 120 130 140
Sr rep1 TCCTTCATGA TGTTGAAACT CCTATCTGCT GTACAAACTA TCTTCCTACT GCCCTTCTGA -TTCCATGTA
Sr rep2 ....G -.......A.
Sr rep3 ...C.T.C.. ....C... ..G......G G.C.....A.
Sr rep4 ....G -.........
Sr rep5 ....A ....G T.C.....A.
Sl rep1 C.....-.AG G.C.AC.G.. T.AGCTGC.A ..T.C..... ...CT..TA. ..T....A.G T.C...A...
Sl rep2 C.....-.AG G.C..C.G.. GAAGCTGC.A ..T.C..... ...CT..TA. ..T....A.G T.C...A...
Sl rep3 C.....-.AG G.C.AC.G.. T..GCTGC.A ..T.C..... ...CT..TA. ..T....A.G T.C...A...
Sl rep4 C.....-.AG G.C.AC.G.. T.AGCTGC.A ..T.C..... ...CT..TA. ..T....A.G T.C...A...
Sl rep5 C.....-.AG G.C.AC.G.. T.AGCTGC.A ..T.C..... ...CT..TA. ..T....A.G T.C...A...
Sl rep6 C.....-.AG G.C..C.G.. GAAGCTGC.A ..T.C..... ...CT..TA. ..T....A.G T.C...A...
Sl rep7 C.....-.AG G.C.AC.G.. T.AGCTGC.A ..T.C..... ...CT..TA. ..T....A.G T.C...A...
Sl rep8 C.....-.AG G.C.AC.G.. T.AGCTGC.A ..T.C..... ...CT..TA. ..T....A.G T.C...A...
Sl rep9 C.....-.AG G.C.AC.G.. T.AGCTGC.A ..T.C..... ...CT..TA. ..T....A.G T.C...A...
Sl rep10 C.....-.AG G.C.AC.G.. T.AGCTGC.A ..T.C..... ...CT..TA. ..T....A.G T.C...A...
150 160 170 180
Sr rep1 TTTTTGTCCC TAAT--CTTT TTTTTCCTAC CACGCAAGTG AT
Sr rep2 ......--T. CCTA--A.C. ....C.. ....
Sr rep3 .....TGT.. CCTA--A.C. ....C.. ....
Sr rep4 .....TGT.. CT.A--TC.. ....
Sr rep5 ......-T.. CCTA--A.C. .... --
Sl rep1 .C..G....T A..ATTA.CC ..C.AT..GT GC.......- --
Sl rep2 .C..G....T A..ATTA.CC ..C.AT..GT GC........ T-
Sl rep3 .C..G....T A..ATTA.CC ..C.AT..GT GC........ T-
Sl rep4 .C..G....T A..ATTA.CC ..C.AT..GT GC........ T-
Sl rep5 .C..G....T A..ATTA.CC ..C.AT..GT GC........ T-
Sl rep6 .C..G....T A..ATTA.CC ..C.AT..GT GC........ T-
Sl rep7 .C..G....T A..ATTA.CC ..C.AT..GT GC........ T-
Sl rep8 .C..G....T A..ATTA.CC ..C.AT..GT GC........ T-
Sl rep9 .C..G-...T A..ATTA.CC ..C.AT..GT GC........ T-
Sl rep10 .C..G....T A..ATTA.CC ..C.AT..GT GC........ T-
B
10 20 30 40 50
Sr rep1 KSCC*V*RIV LYLLLWQL** *KSFMMLKLL SAVQTIFLLP F*FHVFLSLI FFFLPRK*
Sr rep2 ....*.*... ....** *......... .... .W..E..... ..PTTQV
Sr rep3 ..R.*G*... ......E.** *..LT..... ....P.R .WVP*IFCPL I..PTTQV
Sr rep4 ....*.*... ...P....*C *......... .... .W....FVPN L......*
Sr rep5 ....*.**.. ....** *......... .... .W.P*IFVP* S......
Sl rep1 -------XWS STTASGNESK RSPSRATASA ASSNYLSYAL QVPKYLGPK. ILLSVPQ
Sl rep2 .A.YSFNDWS STTASSNESK RSPSRAAAEA ASSNYLSYAL QVPKYLGPK. ILLSVPQV
Sl rep3 .A.YSFNDWS STTASGNESK RSPSRATASA ASSNYLSYAL QVPKYLGPK. ILLSVPQV
Sl rep4 .A.YSFNDWS PPYYCIW*RK *.KPF*GYSF .CQFQLS..C SSGSQVSWS* NYPSICAAS
Sl rep5 .A.YSFNDWS PTTASGNESK RSPSRATASA ASSNYLSYAL QVPKYLGPK. ILLSVPQV
Sl rep6 .A.YSFNDWS STTASSNESK RSPSRAAAEA ASSNYLSYAL QVPKYLGPK. ILLSVPQV
Sl rep7 .A.YSFNDWS STTASGNESK RSPSRATASA ASSNYLSYAL QVPKYLGPK. ILLSVPQV
Sl rep8 .A.YSFNDWS PTTASGNESK RSPSRATASA ASSNYLSYAL QVPKYLGPK. ILLSVPQV
Sl rep9 .A.YSFNDWS PTTASGNESK RSPSRATASA ASSNYLSYAL QVPKYLVLKL S.Y.C..C
Sl rep10 .A.YSFNDWS PTTASGNESK RSPSRATASA ASSNYLSYAL QVPKYLGPK. ILLSVPQV
