# Supplementary figures and images for: Mitochondrial genomes of two Sinochlora species (Orthoptera): novel genome rearrangements and recognition sequence of replication origin
Source: BMC Genomics. 2013 Feb 20;14:114. doi: 10.1186/1471-2164-14-114 (PMC3630010; doi:10.1186/1471-2164-14-114)

# A

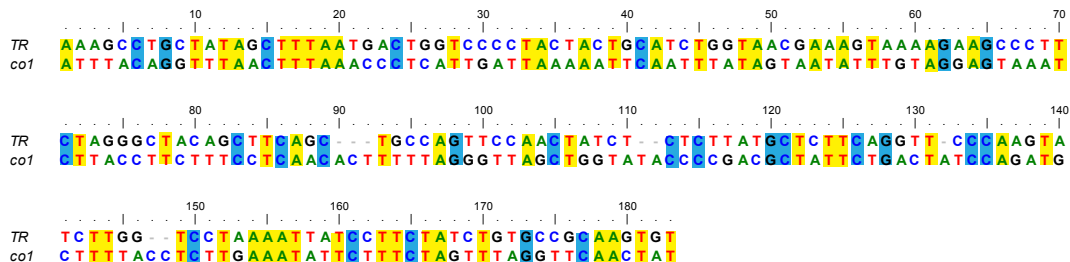

# B

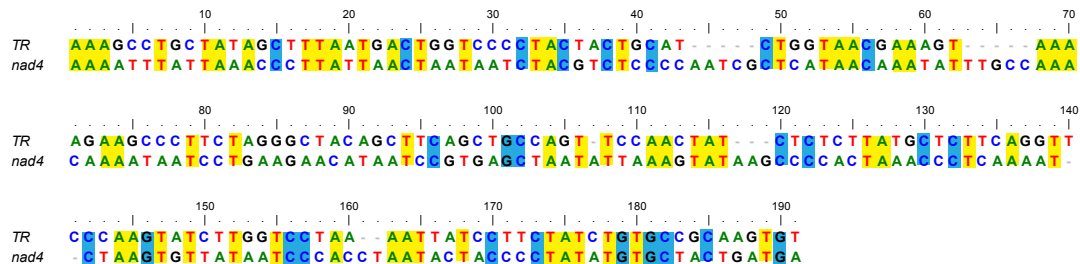

Supplement: Additional file 5 — Alignment of the ORF sequences with cox1 (A) and nad4 (B) in Sinochlora longifissa. Both cox1 and nad4 sequences are from the J-strand. Conserved A and T bases are highlighted in yellow boxes, whereas conserved G and C bases are in blue. [file 1471-2164-14-114-S5.pdf]

A

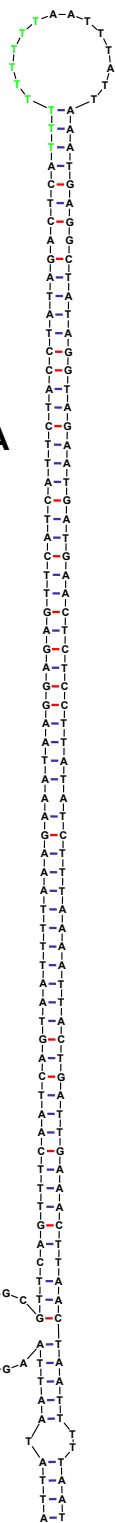

B

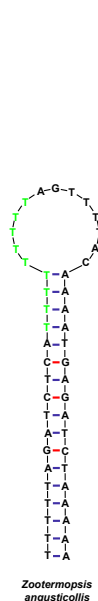

Macrognothotermes errator

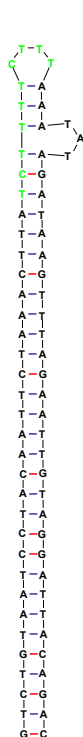

Coptotermes formosanus

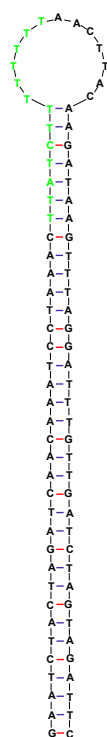

Heterotermes sp.

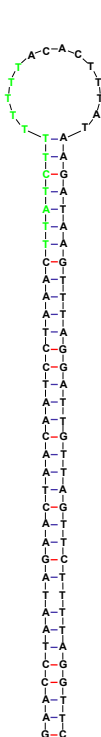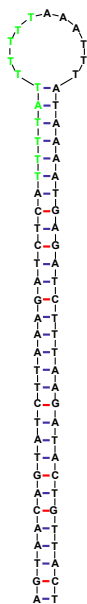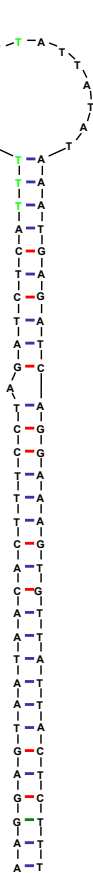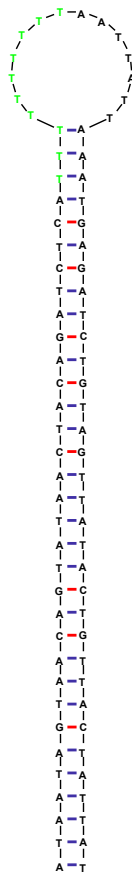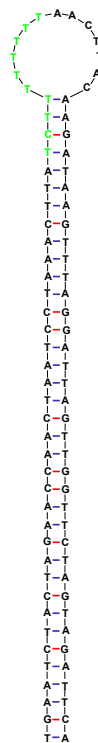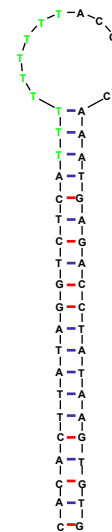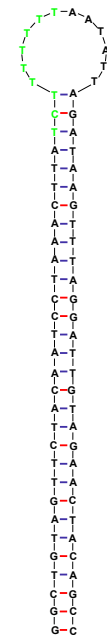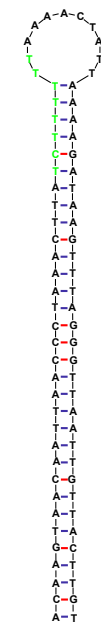

Supplement: Additional file 8 — The potential stem-loop structure involving a T-stretch or T-stretch variant in cockroaches (A) and termites (B). The nucleotides highlighted in green represent the T-stretch variant. [file 1471-2164-14-114-S8.pdf]
