# Supplementary material for: Mitochondrial genomes of two Sinochlora species (Orthoptera): novel genome rearrangements and recognition sequence of replication origin
Source: BMC Genomics. 2013 Feb 20;14:114. doi: 10.1186/1471-2164-14-114 (PMC3630010; doi:10.1186/1471-2164-14-114)
Supplement: Additional file 6 — Alignment of the non-coding spacer between trnS(UCN) and nad1. The 7-bp conserved motif (THYTHDA) across Orthoptera is boxed. However, the motif is not present in Mekongiella xizangensis. [file 1471-2164-14-114-S6.pdf]

Chorthippus chinensis	ATTAA	TTCTAAA	AAAATTTCACTAAATATATGAAATCAATAAAATCTTAAA ▪▪▪
Atractomorpha sinensis		TTCTCAA	AAAATTTCA
Schistocerca gregaria gregaria		TTCTCAA	AAAATTTCA
Calliptamus italicus	TTTAA	TTCTCAA	AAAATTTCA
Acrida willemsei	TAAAA	TTCTAAA	AAAAATTAA
Locusta migratoria	TTAAA	TTCTTAA	AATTTAA
Phlaeoba albonema	TA	TTCTTTA	AAAAATTAA
Oedaleus asiaticus	TTAAA	TTCTTAA	AAAATTTAA
Gastrimargus marmoratus	TTTAAA	TTTTTTA	AGAATTTAA
Oxya chinensis	TTCTA	TTCTAAA	AAAATTTAA
Mekongiella xizangensis	TAGTTA	TTCTTAA	AAAATTTCA
Mekongiana xiangchengensis			CA
Euchorthippus fusigeniculatus	ATTAA	TTTTTAA	AAAATTTCA
Gomphocerippus rufus	A	TTCTAAA	AAAATTTCA
Gomphocerippus licenti	ATTAT	TTCTAGA	AAAATTTCA
Traulia szetschuanensis	GTAAA	TTCTTAA	AAAATTTCA
Arcyptera coreana	ATAAA	TTCTAAA	AAAATTTAATGTACAAAATTTATTTCA
Physemacris variolosa	TGTAAACATTAA	TTCTTAA	AAAAATTAATTA
Xyleus modestus	AAAAATAATTTTAAAT	TCCTATA	AATTTTTAACAATTCTAAAAAGAAATTAAGTTAATTAAA ▪▪▪
Anabrus simplex		TACTAAA	TTAAATCCA
Deracantha onos	T	TACTAAA	TTTAATGCA
Gampsocleis gratiosa		TACTAAA	TACAATACA
Gryllotalpa orientalis	CGTAAACTACATTT	TACTAAA	TATATATCAAAAA
Gryllotalpa pluvialis	AAAATAACATTT	TACTAAA	TATATATCAAAAA
Myrmecophilus manni	GTTATCTT	TACTAAA	AAAATCTCTGAACCCTCCCTACAAAAGTTAGAATTTCAAACC
Ruspolia dubia	C	TACTAAA	ACATTTACATTAAA
Teleogryllus emma	ATT	TCCTAAA	ATGTCTCAATAAAC
Troglophilus neglectus	A	TACTTAA	AATAATTCA
Elimaea cheni		TACTAAA	TTTAATGCA
Sinochlora longifissa	▪▪▪ CTCATATGTTATT	TACTTAA	ATTAATTCAC
Sinochlora retrolateralis	▪▪▪ AGTACAATTAACT	TACTTAA	ATTAATTCAC
