# Supplementary material for: Mitochondrial genomes of two Sinochlora species (Orthoptera): novel genome rearrangements and recognition sequence of replication origin
Source: BMC Genomics. 2013 Feb 20;14:114. doi: 10.1186/1471-2164-14-114 (PMC3630010; doi:10.1186/1471-2164-14-114)
Supplement: Additional file 7 — Comparison among the sequences in the control region of the template strands in Orthoptera. The portion is next to the rrnS gene except that in the two Sinochlora species the sequences are the portion that is next to nad2. Location of the free 5’ ends marking the ON of L. migratoria [4] is indicated. Large arrowheads indicate the sites where major signals were observed and small arrowheads show the sites where minor signals were observed. Arrows indicate the direction of replication. The nucleotide sequence of L. migratoria, which potentially forms the stem-loop structure upstream of the ON, is underlined. The nucleotides highlighted in red represent the location of T-stretch or T-stretch variant.) [file 1471-2164-14-114-S7.pdf]

L. m. migratoria 5' TGGAACTCTTATTCCAATAAATGTATTATAGTATCTTTCTTT--ATTATTTAATCTTTCTTTTCACT-----AATAATAAAGAAAGATTAAATAATATAA3'

L. migratoria TGGAACTTTTATTCCAATAAATGTATTATAGTATCTTTCTTT--ATTATTTAATCTTTCTTTTCACT-----AATAATAAAGAAAGATTAAATAATATAA

G. marmoratus TGGAACTTGTATTCCAATAAATGTATTATAGTATCTTTCTTT--ATTATTTAATCTTTCTTTTCACT-----AATAATAAAGAAAGATTAAATAATATAA

O. asiaticus TGGGTCTTACATTCAGTTAAATGTATTATATTATCTTTCTTT--ATTATTTAATCTTTCTTTTCACT-----AATAATAAAGAAAGATTAAATAATATAA

A. willemsei TGGTATATGTATCCTAATAAGTGGTTTTTATAATCTTTCTTT-CTTTATTTAATCTTTCTTTCTACT-----AATAATAAAGAAAGATTAAATAATATAA

C. italicus TGGTACTTGTATTCGGATAAATGTTTTATATTATTATTTTTATCATTTAATCTTTCTTTTTTTTTT------AATATTAAAGAAAGATTAAATAATATAA

O. chinensis TGGTACTAATATTACAATAAATGTTTTGTAT-ACTATATGTAT-ATTTAATCTTTCTTTTTTTTTT----ATATTTACAAAGAAAGATTAAATAATATAA

P. albonema TGGAACTTGTATCCTGGTAAATGTTTTATTCTCTTTCTTTTCTTTATTTAATCTTTCTTTCTACT-----AACTAAATAAAGAAAGATTAAATAATATAA

S. g. gregaria TGGAACATGTATAGCAATAAATGTATTATAT--TCTTTCTT------ATTTAATCTTTCTTTTCACT----AATAAATAAAGAAAGATTAAATAATATAA

O. longipennis TGGAACTAAAAAAATAAAAAATGTGTAACTCTATG-CTTCTATT-ATTTAATCTTTCTTTTTTTTTT----ATATTTATAAAGAAAGATTAAATAATATAA

E. fusigeniculatus CGGATCAAATATGAGCTGCAATGTTTTAAATCTTTCCT-CCTATATTTAATCTTTACCTTTTTTTT-----AACTATATGGTAAAGATTAAATAATATAA

G. licenti TGGATCAAACAT---GAAAAGGGTTGTTTATTTCC--CCTATATTTAATCTTTACCTTTTTTT--------AACTATATGGTAAAGATTAAATAATATAA

C. chinensis TGGATCAAATAT---GATAAGGGTTGTTTATTTCT--CCTATATTTAATCTTTACCTTTTTT---------AACTATATGGTAAAGATTAAATAATATAA

C. parallelus TGGATCCG-TATAAAACGGAATGTTATAAATTATTCTTT-CCTATATTTAATCTTTACCTTTTT-------AACTATATGGTAAAGATTAAATAATATAA

G. rufus TGGATTCTATATCTGGTGGAATGTTTTAAATTATCCTTTCCTATATTTAATCTTTACTTTCTTT------GACTATATGGTAAAACTTAAATACCGTTA

P. arctica AGAATTCAATAATGAATCAAATTTAGTTCTAGGTTACTTTTATTC-TATTTAATATTTTTTTATATTC----------TAAAAAAGATTAAATAATATAA

T. szetschuanensis AGAATTCA-TCGATTAACGAATTTCTAGACCTAAAGTAGTTTTTATTCCATTTAATCTTTTTTTTTTTTT--ATATCTTAAAAAAGATTAAATAATATAA

A. coreana TGAATTCA---ACAAAGGGATAGTATTGCTTAGAATAGTCTT-ATT--CATTTAATCTTTTTTTTTTTT-ATACTTCCAAAAAAAGATTAAATAATATAA

M. xiangchengensis TGGAACTAAAAT-AAAAAAATTGTGTAATTCTATGCTCCTAT--TATTTAATCTTTCTTTTTTTT---ATATATTTATAAAGAAAGATTAAATAATATAA

A. sinensis TGGAACTTGTATCCTGGTAAATGGTTTTTATTCTTTTATTCTATTATTTAATCTTT--CTTTTCACT-----AATTATAAAGAAAGATTAAATAATATAA

M. xizangensis AATAATTAATCATACGATTGATTTCTGTTCTAATCAAGTGTTAGTGTATTTAATCCTTCTTTTTTTTTT----ACTATAAAGGAGGATTAAATAATATAA

X. modestus TAAAATTATTCAAATAAGACTGCTTTAGTCTAGACTAGAGTAATTTCTCATTTAATCTTTTTTTTTTTTTTTTC--AAAAAAAAAGATAAAAAAAGATCG

T. schrenkii TGGTACCTCTATCAATCAGAATGTTTTATATTATTCTAGTCTATATTTAATCTTTACCTTTTTTT-----ATATTATATGGTAAAGATTAAATAATATAA

P. variolosa ACCCTTAAACCAAAAAAATATTTAAAAAATTCTAA--AATAGATTAAAATCTCTTTCTCCACACTTCCCCCCTTACTTATAGTCAATATAAATCTTAAAT

E. minuta TTTTAGTTAATAAAAAAAAGTAATAATCCCCCCCCTCCCTCCGGTCGYCCCTCTCTCCCTCTTAGGATTCTTTTTTTTTTTT--AGCTCTAAAAAAAAAA

G. orientalis TAGAGGATTAGAGGATATTGTTTCATAACCTTTATATTACATAGATTATTGCATTTTTCTCTTTTTTTTATGCTTTT--AAAAGAGAAAAATGCTTTGCA

G. pluvialis TAGAGGATTAGAGGATATTGTTTAATAATCCTTATATTACATAGATTATTGCATTTTTCTCTTTTTTTTATGCTTTT--AAAAGAGAAAAATGCTTTGTA

M. manni CGAGAAAATTGTGTTAATTATCCTATCTCTTTAAAACCCCTTTGTGATTTTTTTTCTTTCTTTTTTATTCCTC-----AAAGAAAGAAAAAAAATTACAC

T. emma ACAATCATTAAAAAGAACAATACTATTAATACTATACCTAATTTTTCCTCTTTTTTTTTTCT---------AGCAAAAAAGGAAAAATTATCGGTGATGA

G. firmus TTACATTACTTTCAATAAACTGATATAAATTAATATTACTACTAATTTTTCCTTTTTTTTTTTTTTTCTT---ACAAAAAAAGAAAAATTATCTTTTATGA

S. retrolaterialis TAAATTTTAGTCCATAAAAAGAATATT--ACTTTAGTAAGAAAGTAAATTTTTTTTTTTTTTTTTTTTTTATTGATTAATTCCTTCTTGATCTACATTTAA

S. longfissa TTAATTTTAGTAACGATAAAAAATCTTTTTATTTAGTAAGAA-GTAAATTTTTTTTTTTTTTTTTTTAT-ACTGATTAATTCCCTCTTGATCTACATTTAA

A. simplex TTCAGCATCGGGTAATAAATATTTAATTAGAATACTAATCATAGTATATTTTTTTTTTTTTTTTTTTTGTGTATATTTCCTGAGATATATACTTAAATAG

D. onos GAGCCGCCCCCGCCCCGTAATAATAGTTTAATGTAATAGTTAATTATATTTTTTTTTTTTTTTTTTTTTGCGTAGGATTTTTCCTACTAATAATATACTTT

E. cheni TTTGATAAATATCTATATAGCTTTAATAACAATTTAAAATCGAAGGGAAGGAGTTTTTTTTTAGATATTTGCATTCCGGAATATCTTCATCTACGTATT
